# Extensive left atrial thrombus invading pulmonary veins and mitral valve in severe mitral stenosis

**DOI:** 10.12669/pjms.42.7.13395

**Published:** 2026-07

**Authors:** Khola Jamila, Khuzaima Tariq

**Affiliations:** 1 Dr. Khola Jamila Postgraduate Resident, Cardiothoracic Surgery Department, NICVD Karachi, Pakistan; 2 Khuzaima Tariq Assistant Professor, Cardiothoracic Surgery Department, NICVD Karachi, Pakistan

**Keywords:** Anti-coagulation, Atrial fibrillation, Thrombus, Left atrium, Mitral valve Stenosis, Thromboendarterectomy. Thrombectomy

## Abstract

Severe mitral stenosis is a valvular heart disease that obstructs blood flow from the left atrium to the left ventricle, leading to impaired ventricular filling, left atrial dilatation, atrial fibrillation, and thromboembolic events. We report the case of a female patient who presented with shortness of breath (NYHA Class-II) for six months. On examination, a low-pitched murmur was heard at the apex; other systemic examination findings were unremarkable. TTE/TEE revealed left atrial dilatation with LAVI of 120 ml/m^2^, a large left atrial thrombus, severe rheumatic mitral stenosis, and moderate mitral regurgitation with preserved systolic function, leading to MVR. The thrombus was firmly adherent; after failed attempts to create a plane, it was successfully removed using a polypropylene needle. The unusually small mitral annulus was managed by creating fenestrations. This case highlights the importance of preoperative evaluation and individualized surgical approaches for improved outcomes.

## INTRODUCTION

Mitral stenosis is an acquired disease that obstructs blood flow from the left atrium to the left ventricle, leading to left atrial pressure overload and chamber dilatation. This promotes the occurrence of AF and left atrial thrombus formation.^1^ The incidence ranges from 7% to 38% in cases of left atrial enlargement associated with mitral stenosis and atrial fibrillation.^2^ Left atrial dilatation promotes blood stasis and turbulence, increasing the risk of thromboembolism and sudden death.^3^ To carefully remove the organized thrombus while preserving the integrity of the left atrium and surrounding structures, a clear strategy is needed in case the usual maneuvers initially fail. This case demonstrates how tailored surgical techniques can enhance the safety and success of managing such rare presentations. It also highlights the importance of thorough preoperative assessment, timely anti-coagulation, and team based decision making prior to surgery.

## CASE PRESENTATION

A 48 years old woman presented to the emergency department in November 2023 with a syncopal episode and shortness of breath (NYHA Class-II) for six months. She was a known case of neurosarcoidosis for seven years, with no family history of cardiovascular disease and no conventional risk factors such as diabetes, hypertension, or dyslipidemia. On detailed clinical and cardiovascular examination, a low-pitched murmur preceded by an opening snap was detected at the apex, while other systemic examinations were unremarkable.

**Table-I:** Summary of preoperative transthoracic echocardiographic findings.

Parameters	Value	Normal ranges	Interpretation
Left atrial volume index	120 mL/m²	<34 mL/m²	Severely dilated
Mitral valve area	0.6 cm^2^	4–6 cm²	Severe stenosis
Mean pressure gradient	10mmHg	<2 mmHg	Elevated
LVEF	55%	55–70%	Normal
PASP	30mmHg	<35 mmHg	Normal/Underestimated

Based on the clinical presentation of syncope and exertional dyspnea, along with the finding of an apical diastolic murmur with opening snap, the initial differentials included rheumatic mitral stenosis, left atrial myxoma, and intracardiac thrombus.

Considering the patient’s presenting symptoms, TTE/TEE was performed and showed severe mitral stenosis. The patient was managed medically with advice for follow-up in six months due to initial clinical stability. She presented again with worsening shortness of breath, paroxysmal nocturnal dyspnea, and palpitations, but no syncope, dysphagia, or hoarseness. TEE revealed a left atrial thrombus with atrial fibrillation, and she was started on anti-coagulation therapy with an INR maintained within therapeutic ranges.

Later, she developed two syncopal episodes likely due to hemodynamic compromise, and repeat TTE/TEE revealed a severely dilated left atrium (LAVI 120 ml/m²), a large left atrial thrombus, severe rheumatic mitral stenosis (MVA 0.6 cm², mean pressure gradient 10 mmHg), preserved systolic function, moderate tricuspid regurgitation, and normal pulmonary artery systolic pressure (30 mmHg). Following repeat TTE/TEE demonstrating progression to severe rheumatic mitral stenosis with a large left atrial thrombus, the case was discussed in a multidisciplinary team (MDT) meeting, where surgical mitral valve replacement with left atrial thromboendarterectomy and left atrial appendage ligation was planned.

After median sternotomy and commencement of CPB, mitral valve was approached through the LA groove of Sondergaard’s. The cavity was extensively filled with organized thrombus, extending into all four pulmonary veins and encroaching upon the mitral valve leaflets. The thrombus was firmly adherent to the left atrial roof and walls, making thromboendarterectomy particularly challenging. Initial attempts to find a dissection plane using Watson Cheyne dissector and later a curette were unsuccessful. Eventually, the thrombus began to separate with the use of a 4/0 Polypropylene needle, allowing the development of a plane. The left atrial appendage (LAA) was ligated, anterior mitral leaflet (AML) was excised, posterior leaflet was partially preserved with fenestration to accommodate the prosthetic valve in a small annulus (25 mm), allowing placement of a 27 mm bileaflet mechanical prosthetic valve. The patient was weaned off bypass with the pacemaker on backup. Three days later, a permanent pacemaker (PPM) was implanted for heart block, which was anticipated due to extensive mitral valve dissection. Follow up TTE was done after six months which showed moving discs and normal gradients across the valve.

**Fig.1:**
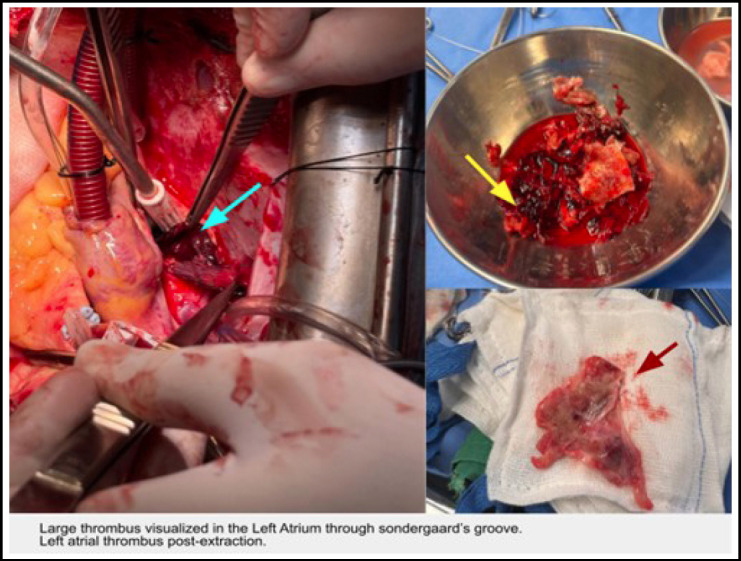
Thrombus after removal during surgery.

## DISCUSSION

In chronic mitral valve disease with obstruction, the left atrium often enlarges, leading to increased pressure and volume within the chamber. This dilation helps relieve pulmonary congestion and prevents pulmonary hypertension and edema at the cost of atrial fibrillation, as evident in a patient who had normal pulmonary arterial pressures and a giant left atrial cavity.^4^ Left atrial size increases due to obstruction of blood flow, as seen in mitral stenosis. Studies by Niaz et al. have demonstrated an inverse relationship between mitral valve area and left atrial size, suggesting that a smaller mitral valve area is associated with greater left atrial dilatation. This finding is consistent with an unusually small mitral valve area associated with a giant left atrium, reinforcing previously reported observations.^5^ As reported in previous studies, combined mitral stenosis and mitral regurgitation can lead to marked left atrial dilatation. Despite a significantly enlarged left atrium, this patient had no compressive symptoms such as dysphagia, hoarseness of voice, or lung collapse, and no signs of right heart failure, consistent with findings by Hoque A et al. and others. This is an unusual presentation for such advanced disease.^6^

**Fig.2:**
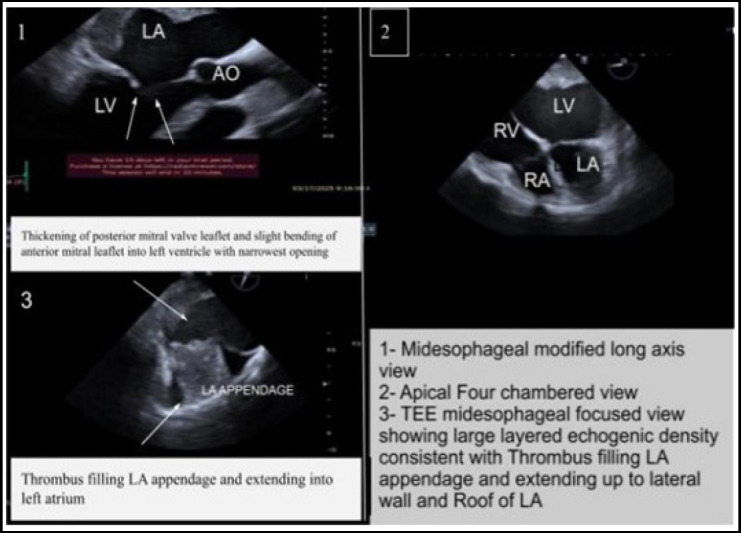
Thrombus involving LA in pre operative TOE images.

An enlarged left atrium along with this disease are well-known to create a highly thrombogenic environment by causing sluggish blood flow and disrupting the integrity of the atrial endocardium, increasing the risk of thrombus formation. In patients in sinus rhythm, anti-coagulation may be considered when TTE or TEE shows marked spontaneous echo contrast or significant left atrial enlargement (M-mode diameter >50 mm or left atrial volume >60 mL/m²), consistent with a Class-II(A) recommendation and Level of evidence.^7^ Atrial fibrillation further increases the risk of clot formation compared with sinus rhythm, often progressing from spontaneous echo contrast to organized thrombus. In this case, the thrombus was friable and fragmented during removal, reflecting its fragile nature, although no embolic complications occurred.^8^

A study by Vogiatzis I et al. reported complete resolution of a left atrial thrombus in patients with mitral stenosis and atrial fibrillation using intravenous unfractionated heparin along with oral anticoagulants. Thrombolytic therapy has also been used for large thrombi in previous studies. Therefore, the choice between surgical thrombectomy and medical treatment with heparin or thrombolytics along with anti-coagulation remains debated in these cases.^9^ Anti-coagulation was done with VKA – Tab Warfarin 5-mg as per guidelines, which showed no radiological evidence of thrombus regression. However, such therapeutic approaches, if considered preoperatively, may facilitate the reduction or resolution of a large organized thrombus, potentially aiding in its surgical removal when extraction is challenging.

Various surgical approaches to the left atrium can be used to address these challenges. The standard approach through the Sondergaard’s groove provided direct access to the mitral valve and left atrial cavity, making thrombectomy relatively easier. A biatrial incision may allow en bloc thrombectomy; however, complete extraction is often not possible when the thrombus is firmly adherent with no clear plane. Complete removal was achieved via the standard approach, and no pericardial patch was required. In some cases, pericardial patch repair may be needed to exclude residual thrombus and reduce the risk of recurrence.^10^ Delayed diagnosis of mitral stenosis due to limited access to advanced imaging and patients’ tendency to ignore early symptoms significantly increases surgical risk and predisposes to serious postoperative complications.^11^ This was reflected by the development of complete heart block following extensive mitral valve dissection and thromboendarterectomy.

## CONCLUSION

Severe mitral stenosis with a giant left atrium significantly increases the risk of atrial fibrillation, which further contributes to chamber dilatation, blood stasis, and thrombus formation. However once formed, thrombus regression remains unlikely despite effective anticoagulation. The combination of a fragile, extensive thrombus and an unusually small mitral annulus presented a rare and life-threatening surgical challenge.
